# The dilemma of partial nephrectomy and surgical upstaging

**DOI:** 10.1590/S1677-5538.IBJU.2021.0859.1

**Published:** 2022-08-03

**Authors:** Rodolfo Borges dos Reis, Rafael Neuppmann Feres, Marcelo Cartapatti da Silva, Valdair Francisco Muglia, Antonio Antunes Rodrigues

**Affiliations:** 1 Universidade de São Paulo Faculdade de Medicina de Ribeirão Preto Departamento de Cirurgia e Anatomia Ribeirão Pretom SP Brasil Departamento de Cirurgia e Anatomia, Faculdade de Medicina de Ribeirão Preto, Universidade de São Paulo, Ribeirão Pretom, SP, Brasil;; 2 Universidade de São Paulo Faculdade de Medicina de Ribeirão Preto Departamento de Imagens Médicas, Radioterapia e Oncohematologia Ribeirão Preto SP Brasil Departamento de Imagens Médicas, Radioterapia e Oncohematologia, Faculdade de Medicina de Ribeirão Preto, Universidade de São Paulo, Ribeirão Preto, SP, Brasil

## COMMENT

Malignant kidney neoplasms are increasingly on the rise. The National Cancer Institute expects, in 2022, in the United States, 79.000 new cases and 13.920 deaths. Precise clinical staging at diagnosis impacts therapy, prognosis, and oncologic outcomes.

The TNM (Tumor-Node-Metastasis) staging system was initially proposed by the French surgeon Pierre Denoix, between 1943 and 1952, at Institute Gustave-Roussy. To this day is considered a tool to estimate solid tumor prognosis (1, 2).

TNM is rooted in the Halstedian principle of temporal determinism that solid tumors spread sequentially from the primary site to lymphatics, then to distant organs. TNM clinical staging is based on the anatomic spread of the disease. However, the TNM system has several drawbacks:

1. It is limited by the need for a correlation between the anatomic progression of the disease and the progression to more advanced stages. Furthermore, in patients with equivalent anatomic spread, heterogeneity is induced when variable outcomes (recurrence or survival) are forced into the same stage.
2. It fails to incorporate the variables: tumor, nodules, and metastases as continuous variables. This fact creates a system with a finite number of stages, limiting the determination of an individual prognosis.
3. It relates a clinical outcome (prognosis) to descriptive, not determinant, variables. It states that if the disease is anatomically more advanced the prognosis will be worse, without considering other variables, such as biomarkers, genetic scores, histology, and behavioral factors.

Nomograms are visual graphical representations of equations that allow clinicians to estimate the probability of a final medical outcome. It uses a points-based system whereby patients accumulate points based on levels of the selected variables.

Nomograms are widely used in different clinical scenarios and have become an epidemic in the recent medical literature. The search of articles in PubMed using the term “cancer nomogram” retrieved 8344 articles between 2012 and 2022. In oncology, they are commonly used to estimate the risk of recurrence and death. Nomograms incorporate anatomic and non-anatomic variables from a specific patient, resulting in a personalized and more precise tool to risk assessment (3).

In recent years we observed the development of new nomograms incorporating old and new prognostic and risk factors (genetic, biomarkers, histology). Although, the vast majority are not validated in different populations (4-7).

The inclusion of the TNM classification as a variable in the majority of the new nomograms corroborates its importance. TNM is an anatomic classification, and the accuracy of imaging methods is crucial to reduce it as low as possible surgical upstaging.

Imaging is typically performed using contrast-enhanced CT, although there is a risk of missing renal sinus fat invasion, perirenal fat invasion, or renal vein thrombosis, which can lead to pT3a upstaging (8-10). In 2020, Veccia A. et al. published a systematic review and meta-analysis of the outcomes and predictive factors for upstaging to pT3a in 21869 patients who underwent partial or radical nephrectomy for cT1 renal tumors. The authors concluded that upstaging is not common, but was correlated with worse oncologic outcomes. Upstaging was correlated to age, tumor size and complexity, and histology. (11, 12) de la Barra CC et al. reported a preoperative model to predict pT3 upstaging in renal cancer. The authors developed a nomogram that included age, contact with the main vessels, and size. The nomogram presented an AUC (area under the curve) of 0.864 in the ROC curve (13).

The study by Cao et al. published in the International Brazilian Journal of Urology (14), addressed the need for a tool to help urologists predict upstaging for localized cT1 renal cancer. The authors evaluated retrospectively 2712 patients with Renal Cell Carcinoma and cT1a disease, 121 (4.5%) were upstaging to pT3a on the final pathology report. Based on the findings, they constructed a nomogram with the variables, age, tumor size, maximum and minimum diameter ratio, and fibrinogen level. They split the whole population in two, one for validation purposes.They reported the C-index for predicting upstaging (cT1 - pT3a) of 0.756 (95% CI, 0, 6081-0.831), in the validation cohort, the C-index was 0.712 (95% CI, 0.638-0.785). They concluded that their nomogram is a tool for predicting upstaging, cT1 - pT3a, in patients with RCC. They also pointed out the need for multicenter studies to confirm their findings. I congratulate the authors for their work and methodology. Should be highlighted the inclusion of fibrinogen level as a variable. I also agree that the nomogram should be tested in a larger population to confirm or not the findings. Furthermore, adding new variables to the described nomogram may increase its accurancy (C-index).
